# Protective role of mucosa-associated invariant T cells in sepsis-related liver injury

**DOI:** 10.3389/fimmu.2026.1779656

**Published:** 2026-04-15

**Authors:** Wei Bu, Qiang Ji, Yuhang Yao, Yuping Liang, Xuexue Pu, Nian Liu, Min Shao

**Affiliations:** 1 Department of Critical Care Medicine, The First Affiliated Hospital of Anhui Medical University, Anhui Medical University, Hefei, Anhui, China; 2 Center for Big Data and Population Health of IHM, Anhui Medical University, Hefei, China

**Keywords:** cytokine, flow cytometry, inflammations, mucosal associated invariant T (MAIT) cells, sepsis-related liver injury (SRLI)

## Abstract

**Background and objective:**

Sepsis is a life-threatening condition characterized by increasing global incidence, fundamentally driven by immune dysregulation. The liver, crucial for pathogen clearance and inflammatory modulation during sepsis, frequently exhibits functional impairment. Mucosal-associated invariant T (MAIT) cells bridge innate and adaptive immunity and are integral to antimicrobial defense. Their specific role in sepsis-related liver injury (SRLI) remains unclear. This study aims to elucidate MAIT cell alterations and function in SRLI using clinical cohorts and murine models.

**Methods:**

The cohort comprised 47 SRLI patients, 37 with non-septic acute liver injury (NSLI), 34 patients with non-associated liver injury in sepsis (NLIS), and 115 healthy controls (HC). Peripheral blood MAIT cells were characterized by flow cytometry for frequency, phenotype, and cytokine production. *In vitro* experiments assessed effects of bilirubin and cytokines on MAIT cells. A murine SRLI model was established via intraperitoneal lipopolysaccharide (LPS) injection. Liver injury severity, inflammatory cytokines, and histopathology were compared between wild-type and MAIT cell-deficient mice.

**Results:**

In the SRLI group, the proportion of peripheral blood MAIT cells was significantly decreased compared to the HC group. Regarding activation (CD69^+^, CD25^+^) and exhaustion (PD-1^+^, Tim-3^+^) phenotypes, the expression levels of CD69 and PD-1 were markedly upregulated relative to all other groups, while the expression of CD25 and Tim-3 was increased compared to the HC and NSLI groups. *In vitro* assays indicated that upon stimulation, these MAIT cells preferentially produced IL-17A, TNF-α, and granzyme B, suggesting a bias toward Th17-like differentiation. Elevated concentrations of bilirubin exacerbated both the activation and exhaustion of MAIT cells through a TCR-dependent mechanism. In the septic murine model, MAIT cells deficiency led to more severe liver injury, and higher serum levels of transaminases, bilirubin, and pro-inflammatory cytokines compared to wild-type.

**Conclusion:**

In the context of SRLI, peripheral blood MAIT cells exhibit a diminished frequency, functional impairment, and a phenotypic shift toward a Th17-like profile. Among these, bilirubin plays a key role. Ultimately, MAIT cells exert a protective role in sepsis-induced liver injury by suppressing excessive inflammatory responses.

## 1 Introduction

Sepsis is defined as a life-threatening syndrome characterized by an infection-induced, dysregulated host response that results in organ dysfunction. It represents a significant and critical concern in intensive care units (ICUs) (1). It is estimated that sepsis affects about 1.5 million individuals each year and contributes a high case-fatality rate ranging from 20% to 30% (2). Sepsis is characterized by an initial phase of hyperinflammation, followed by subsequent immunosuppression and progressive organ dysfunction. This condition activates the host immune system, resulting in an inflammatory storm and dysregulated immune responses (3). This cytokine storm, in turn, promotes cellular dysfunction and apoptosis, causing organ failure and death (4). The effective prevention and control of organ damage is imperative for mitigating the burden and mortality risk associated with sepsis. Nonetheless, the mechanisms underlying hepatic inflammatory injury in septic patients, a critical manifestation of multiple organ dysfunction, remain elusive. This knowledge gap underscores the urgent need for comprehensive investigation into the pathogenesis of sepsis-related liver injury (SRLI). As a crucial organ in the human body, the liver modulates immune defense by eliminating bacteria, synthesizing acute-phase proteins and cytokines, and initiating adaptive inflammatory responses (5). Therefore, the gut-liver axis is a pivotal target for intestinal flora in sepsis (6). The systemic inflammatory response, immune dysregulation, and impaired oxygen delivery to hepatocytes caused by sepsis can result in acute liver injury, collectively known as SRLI. SRLI is typically characterized by a total bilirubin level >34.2 μmol/L (2 mg/dL) and coagulopathy with an international normalized ratio (INR) >1.5 (7). Liver dysfunction markedly impairs the prognosis of patients with sepsis and serves as a significant predictor of mortality in the ICU (8). However, the efficacy of currently available clinical therapies remains constrained. Consequently, the early identification of sepsis patients exhibiting concomitant acute liver injury, alongside the implementation of timely and effective interventions, is of paramount importance.

Mucosal-associated invariant T (MAIT) cells represent the most abundant innate-like T cells in humans. These cells recognize microbial riboflavin metabolites presented by the MHC class I-related protein MR1 and operate through a T cell receptor (TCR)-dependent mechanism (9). In the absence of antigen presentation, MAIT cells can also be activated by IL-12, IL-15, and IL-18 in a TCR-independent manner to participate in the immune response against pathogens (9, 10). Upon activation, MAIT cells can rapidly releasing potent immunomodulatory mediators, including TH1-type cytokines such as IFN-γ TNF-α, and/or the TH17-type cytokine IL-17. Additionally, these cells contain granules that house cytotoxic effector molecules, including granzyme B and perforin, which are secreted upon activation. Moreover, MAIT cells express a diverse array of homing receptors, including CCR5, CCR6, CXCR3, and CXCR6 (11, 12). Therefore, MAIT cells can elicit cytotoxic responses leading to target cell lysis under microbial infection conditions in both experimental and physiological contexts (13), and exhibit a robust capacity to home to inflammatory sites (14). This functional repertoire renders them indispensable in responding to infectious challenges.

Phenotypic alterations in innate immune cells, including MAIT cells, are closely correlated with the progression of SRLI, with the liver serving as a primary site of inflammation. The innate immune system constitutes the first line of defense against hematogenous bacterial translocation. Upon reaching the liver via the hepatic artery and portal vein, bacteria and their products (e.g., lipopolysaccharide) are known to activate Kupffer cells, which subsequently produce a plethora of inflammatory cytokines (such as IL-1β, IL-6, and TNF-α) and chemokines (e.g., CCL2). This process facilitates the activation of additional immune cells (including neutrophils and monocytes) and can result in a cytokine storm (15, 16). A study indicated that, in comparison to the sepsis group without liver injury, patients experiencing SRLI demonstrated significantly elevated serum levels of inflammatory markers (IL-6, PCT, LDH), which suggests a more pronounced inflammatory response (17). The involvement of other innate immune cells, particularly innate-like T cells, in this process is not yet fully elucidated. Consequently, this study commences by investigating the phenotypic alterations of MAIT cells during the initiation and progression of SRLI. Additionally, it seeks to explore their complex regulatory functions within the local hepatic immune microenvironment under inflammatory conditions, thereby identifying potential therapeutic targets for this condition.

## 2 Materials and methods

### 2.1 Patient information

Adult patients with sepsis (including those with sepsis or septic shock) admitted to the Intensive Care Unit (ICU) of the First Affiliated Hospital of Anhui Medical University were enrolled in this study from January 2022 to December 2023. The diagnoses of sepsis and septic shock were established according to internationally published guidelines (18). Sepsis was diagnosed based on the Third International Consensus Definitions for Sepsis (SEPSIS-3), which requires: (1) a Sequential Organ Failure Assessment (SOFA) score ≥ 2, and (2) confirmed infection. SRLI was defined based on the Surviving Sepsis Campaign (SSC) guidelines as total bilirubin ≥ 34.2 μmol/L (2 mg/dL) accompanied by coagulopathy, indicated by an International Normalized Ratio (INR) > 1.5 (19, 20). The exclusion criteria were delineated as follows: (1) individuals aged ≤ 18 years; (2) patients discharged within 24 hours of ICU admission; (3) those with incomplete or absent medical records; (4) patients diagnosed with other acute or chronic liver diseases, including chronic hepatitis, viral/autoimmune/toxic/ischemic hepatitis, malignant liver tumors, drug-induced liver injury, alcohol-related liver injury, obstructive jaundice, a history of liver surgery, ongoing chemotherapy, or acute liver injury present prior to sepsis onset. None of the patients included in the study had received corticosteroids or other immunomodulatory agents prior to their enrollment, and the progression of their liver injury showed no clear association with the use of clinical medications. The septic patients were classified into two distinct groups based on the presence of liver injury: a SRLI group and a non-associated liver injury in sepsis (NLIS) group. Additionally, a non-septic acute liver injury (NSLI) group was established, comprising ICU patients who developed acute liver injury as a result of trauma or major cardiovascular surgery, with infection was excluded. Finally, healthy controls were selected from individuals undergoing routine health examinations at the Anhui Medical University Health Examination Center, with the exclusion of those presenting with significant acute or chronic illnesses. Survivors were defined as patients who lived for more than 28 days following SICU admission. All patients received standard treatment in accordance with established clinical guidelines (18). This study was approved by the First Affiliated Hospital Ethics Committee of Anhui Medical University (Approval No. PJ2024-02-24). We conducted the study in strict compliance with the Declaration of Helsinki as revised in 2013.

A total of 115 healthy individuals undergoing routine health examinations were enrolled as healthy controls (HC). Additionally, the study included 34 patients diagnosed with NSLI, 37 patients with NLIS, and 47 patients with SRLI. The comprehensive characteristics of the enrolled patients are delineated in **Table 1**, while the corresponding details for the HC are presented in **Supplementary Table 1**. Among the patients exhibiting SRLI, 91.5% demonstrated positive blood cultures, with the microbiological culture results summarized in **Supplementary Table 2**.

**Table 1:** Patients’ baseline characteristics.

Variable	NSLI (n=34)	NLIS (n=37)	SRLI (n=47)	P value
Age, y	62.56 ( ± 16.25)	58.08 ( ± 15.82)	58.08 ( ± 15.82)	0.310
Male sex, n (%)	19 (55.88)	26 (70.27)	38 (80.85)	0.052
BMI, kg/m²	21.80 [20.31, 23.54]	21.21 [17.91, 26.14]	21.23 [19.47, 24.44]	0.9761
White blood cell (WBC), 10^9^/L	11.26 [8.96, 15.61]	11.55 [7.88, 16.76]	12.59 [7.29, 16.54]	0.975
Neutrophils percentage (NEUT% ), %	86.55 [81.63, 90.50]	85.00 [77.90, 88.40]	87.50 [81.55, 92.95]	0.122
Lymphocyte (LYMPH), 10^9^/L	0.66 [0.50, 1.13]	0.97 [0.46, 1.49]	0.69 [0.41, 1.13]	0.338
Lymphocyte percentage (LYMPH% ), %	6.25 [4.23, 8.35]	7.30 [5.70, 10.90]	4.80 [3.00, 9.30]	0.042
Hemoglobin (HGB), g/L	93.50 [78.75, 101.75]	95.00 [79.00, 101.0]	87.00 [77.00, 102.00]	0.712
Platelet (PLT), 10^9^/L	85.00 [57.00, 126.50]	218.00 [100.00, 305.00]	76.00 [27.00, 129.50]	<0.001
C-reactive protein (CRP), mg/L	107.53 [39.36, 147.83]	74.77 [28.30, 143.00]	90.00 [53.57, 160.00]	0.353
Procalcitonin (PCT), ng/mL	3.19 [1.05, 29.20]	3.48 [0.36, 19.66]	5.44 [2.13, 18.04]	0.329
Albumin (ALB), g/L	31.94 [26.95, 36.00]	33.09 [29.50, 38.25]	32.48 [27.80, 36.50]	0.721
Total bilirubin (TBil), μmol/L	44.07 [35.05, 56.75]	19.80 [12.50, 29.80]	64.7 [43.95, 116.70]	<0.001
Alanine aminotransferase (ALT), U/L	56.00 [38.00, 119.75]	45.00 [24.00, 104.00]	70.00 [33.00, 605.00]	0.057
Aspartate transaminase (AST), U/L	72.50 [62.00, 125.75]	50.00 [30.00, 102.00]	63.00 [49.50, 700.50]	0.026
Alkaline phosphatase (ALP), U/L	84.50 [51.25, 114.50]	109.00 [86.00, 137.00]	116.00 [67.50, 170.50]	0.039
Gamma-glutamyltransferase (GGT), U/L	54.00 [24.00, 80.50]	75.00 [37.00, 124.00]	56.00 [33.50, 119.50]	0.488
Blood urea nitrogen (BUN), mmol/L	19.95 [12.26, 30.42]	15.90 [10.50, 20.60]	17.08 [11.76, 23.69]	0.346
Creatinine (Cr), μmol/L	151.95 [83.88, 220.45]	87.60 [64.10, 150.90]	149.80 [78.30, 222.0]	0.047
Estimates of glomerular filtration rate (eGFR), mL/min/1.73m^2^	38.50 [25.50, 83.25]	84.00 [38.00, 103.00]	44.00 [21.50, 92.00]	0.048
Prothrombin time (PT), s	17.60 [16.23, 19.73]	15.50 [14.60, 16.80]	18.90 [16.55, 21.35]	<0.001
International normalized ratio (INR)	1.51 [1.35, 1.68]	1.22 [1.13, 1.42]	1.58 [1.53, 1.87]	<0.001
Duration of mechanical ventilation, days	11.50 [6.25, 22.75]	9.00 [4.00, 14.00]	14.00 [9.0, 26.00]	0.027
Clinical outcome				
Survival for 28 days, n (%)	17 (50.00)	26 (70.27)	17 (36.17)	0.008
ICU length of stay (ICU LOS), days	18.00 [8.00, 32.75]	14.00 [9.00, 23.00]	20.00 [12.00, 38.00]	0.211

Characteristics of patients included in the study. Data are given as Mean (SD), Median [IQR] or frequency with percentages. P values are based on One-way ANOVA Kruskal-Wallis rank sum test for discrete data.

### 2.2 Sample collection

Peripheral blood samples were collected in EDTA tubes, diluted 1:1 with phosphate-buffered saline (PBS, Servicebio), and subjected to density gradient centrifugation utilizing Ficoll-Paque PLUS (Cytiva) for the isolation of PBMCs. The isolated PBMCs were subsequently washed once with PBS and resuspended in RPMI 1640 medium supplemented with 10% fetal bovine serum (FBS). For surface staining, the cells were resuspended in 1 mL of cold (4 °C) RPMI 1640 medium containing 10% FBS to generate a single-cell suspension. A subset of the cells was utilized for intracellular cytokine staining and nuclear transcription factor staining. Plasma was collected and stored at -80 °C for future cytokine detection.

### 2.3 Flow cytometry

The cells were washed with fluorescence-activated cell sorting (FACS) buffer (2% bovine serum albumin, 0.1% NaN_3_ in PBS) and subsequently incubated with various fluorescent dye-conjugated antibody cocktails at 4 °C for 30 minutes. The following antibodies were used for flow cytometry analysis: anti-CD3-FITC, anti-CD4-BV510, anti-CD8-PerCP/Cy5.5, anti-CD69-PerCP/Cy5.5, anti-CD25-PE/Cy7, anti-PD-1-PE, anti-Tim-3-PE, anti-CXCR3-BV421, anti-CCR6-PE/Cy7, anti-CXCR6-BV421, anti-IL-17A-BV421, and anti-IFN-γ-BB700 (all from BD Pharmingen, USA). The clones, suppliers, and catalog numbers of all antibodies used for flow cytometry will be listed in **Supplementary Table 4**. The NIH Tetramer Core Facility provided biotinylated human/mouse MR1 5-OP-RU or 6-FP tetramer to Prof. Hua Wang. We also thank the MR1 tetramer technology which was developed jointly by Dr. James McCluskey, Dr. Jamie Rossjohn, and Dr. David Fairlie, and the material was produced by the NIH Tetramer Core Facility as permitted to be distributed by the University of Melbourne (21). Dead cells were identified utilizing Fixable Viability Stain 780 (FVS780; BD Biosciences). To evaluate apoptosis, cells were stained with Annexin V-PE and 7-AAD (Procell, China) following the manufacturer’s protocol to determine the percentage of apoptotic cells. Data acquisition was performed on a BD FACSVerse flow cytometer (BD Biosciences), and the resulting data were analyzed employing FlowJo software (version 10.8.1).

### 2.4 PBMC isolation and culture

To evaluate cytokine production, PBMCs were stimulated in RPMI-1640 medium supplemented with 10% fetal bovine serum, utilizing phorbol 12-myristate 13-acetate (PMA, 50 ng/mL)/ionomycin (1 μg/mL) in the presence of brefeldin A (Abisin, China) for 4–6 hours at 37 °C. Following stimulation, the cells were fixed and permeabilized using a Fixation/Permeabilization Kit (BD Pharmingen) for subsequent intracellular staining. For the staining of nuclear transcription factor, PBMCs were fixed and permeabilized with the Transcription Factor Buffer Set (BD Pharmingen). In separate experimental conditions, PBMCs were stimulated with IL-15 at a concentration of 50 ng/mL (Novoprotein Scientific, China) for 24 hour.

### 2.5 RNA isolation and real-time PCR

Total RNA was extracted from isolated PBMCs using a total RNA extraction kit (Takara, Japan). Reverse transcription was performed in a 20 µL reaction volume using the PrimeScript™ RT Reagent Kit with gDNA Eraser (Takara, Japan). Target gene expression was analyzed by real-time PCR using TB Green^®^ Premix Ex Taq™ II (Takara, Japan) on a LightCycler/LightCycler 480 system (Roche Diagnostics). PCR amplification was conducted using cDNA derived from PBMCs as the template, with each sample analyzed in triplicate. The mRNA expression levels were normalized to GAPDH, and relative quantification was conducted employing the comparative ΔΔ^Ct^ method. The qPCR primer sequences used in this study are shown in **Supplementary Table 3**.

### 2.6 Mice and septic model

Wild-type (WT) C57BL/6 mice were purchased from Ziyuan Biotechnology Co., Ltd. (Hangzhou, China). MR1 knockout (Mr1^-^/^-^) homozygous mice on a C57BL/6 background (strain ID: NM-KO-190119) were obtained from Shanghai Model Organisms Center, Inc. (Shanghai, China). Adult WT and Mr1^-^/^-^ mice were intraperitoneally injected LPS (10 mg/kg) or PBS for 24 h, as described previously (22–24), to establish sepsis and control models. Subsequently, all mice were resuscitated with 1 mL of normal saline and returned to their cages for recovery, with warming provided for a duration of 1 hour. And the single-cell suspension of liver lymphocytes was prepared using Percoll density gradient centrifugation. All mice were housed in a specific pathogen-free environment that was temperature- controlled at 20 °C and maintained under a 12-hour light/dark cycle at the Animal Experiment Center of Anhui Medical University, with unrestricted access to food and water. All animal experiments were conducted in accordance with the standards for the humane treatment of laboratory animals established by Anhui Medical University and were approved by the Institutional Animal Care and Use Committee (IACUC) of the university.

### 2.7 Isolation of lymphocytes from mouse liver and MR1 tetramer surface staining

Following anesthesia with isoflurane, mice were euthanized via cervical dislocation. Liver tissues were harvested and mechanically dissociated using forceps. Hepatic lymphocytes were subsequently isolated utilizing Percoll density gradient centrifugation. The resulting cell suspension was washed with RPMI 1640 medium supplemented with 10% fetal bovine serum (FBS), and red blood cells were lysed using an appropriate lysis buffer. From each experimental group, cell aliquots containing 1–2×10^6^ cells were prepared. Cells were initially stained with Fixable Viability Stain 780 (FVS780, BD Biosciences) for 15 minutes at room temperature to distinguish viable cells from non-viable cells. Subsequently, they were incubated with appropriately diluted APC-conjugated MR1 tetramer loaded with 5-OP-RU and anti-CD3-PE/Cy5.5 (BioLegend) for 30 minutes at 4 °C in the dark. Samples were acquired using a BD FACSVerse flow cytometer (BD Biosciences), and the data were analyzed employing FlowJo software (version 10.8.1).

### 2.8 Histological analysis

Mouse liver tissues were fixed in 4% paraformaldehyde and subsequently embedded in paraffin. The tissues were sectioned into 5μm slices and stained with hematoxylin and eosin (H&E) for analysis utilizing light microscopy. Nuclei were counterstained with hematoxylin. Neutrophil infiltration in the liver was evaluated through chloroacetate esterase staining (Sigma, St. Louis, MO). Hepatic macrophages were identified via immunofluorescence staining for the F4/80 marker.

### 2.9 Measurement of ALT, AST and TBIL in mouse serum

The serum samples were collected via retro-orbital bleeding at the time of euthanasia. Serum levels of alanine aminotransferase (ALT), aspartate aminotransferase (AST), and total bilirubin (TBIL) were measured using the Piccolo Xpress system (ABAXIS, Union City, CA, USA).

### 2.10 Multiplex immunoassays

Concentration of cytokines (IL-1β, IL-6, IL-17A, IFN-γ, TNF-α) in serum was quantified using a cytometric bead-based immunoassay (LEGENDplex™ Mouse Inflammation Panel, BioLegend) following the manufacturer’s instructions. Data were acquired using a BD FACSVerse™ flow cytometer equipped with FACSuite™ software (BD Biosciences), and sample analysis was performed using the LEGENDplex™ online data analysis system.

### 2.11 Statistical analysis

Data analysis was performed using GraphPad Prism 9.0.0. Statistical comparisons between study groups were conducted using the Mann Whitney U test or the t-test for two groups. Data correlation was analyzed using Spearman correlation. The Kruskal-Wallis test, in conjunction with Dunn’s multiple comparison test or one-way analysis of variance (ANOVA), was used for multiple groups. P < 0.05 was considered statistically significant (*p < 0.05, **p < 0.01, ***p <0.001, ****p < 0.0001).

## 3 Result

### 3.1 Alterations in the frequency and subpopulations of peripheral blood MAIT cells in patients with SRLI

**Figure 1:**
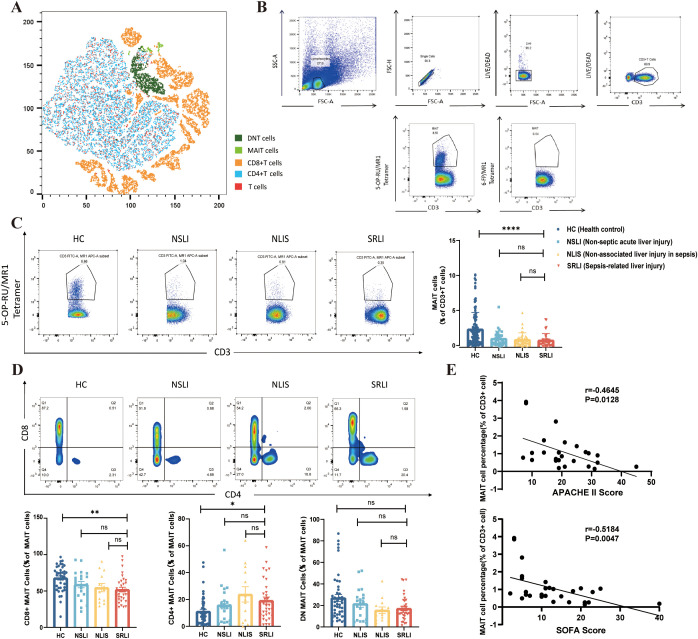
Frequency alterations of peripheral blood MAIT cells in patients with sepsis−induced liver injury. **(A)** T−SNE dimensionality reduction plot showing the distribution of peripheral blood T−cell subsets in sepsis−induced liver injury patients. **(B)** Gating strategy for identifying mucosa−associated invariant T (MAIT) cells. MAIT cells were defined as CD3^+^ MR1−5−OP−RU tetramer^+^ lymphocytes by flow cytometry, with MR1−6−FP tetramer used as a negative control. **(C)** Representative flow cytometry plots and statistical analysis of MAIT cell frequency changes in peripheral blood across groups: HC (n=115), NSLI (n=37), NLIS (n=37), SRLI (n=47). **(D)** Representative flow cytometry plots and statistical analysis of the percentage of different MAIT cell subsets within total MAIT cells across groups: HC (n=40), NSLI (n=21), NLIS (n=13), SRLI (n=33). **(E)** Correlation between SOFA score, APACHE II score, and MAIT cell frequency in sepsis−induced liver injury patients. Ns, not statistically significant; *p<0.05, **p<0.01, ***p<0.001, and ****p<0.0001, indicate statistical significance. HC, Health control; NSLI, Non-septic acute liver injury; NLIS, Non-associated liver injury in sepsis; SRLI, Sepsis-related liver injury.

As shown in **Figure 1B**, this study employed MR1 tetramers loaded with the pyrimidine intermediate 5-(2-oxopropylideneamino)-6-d-ribitylaminouracil (5-OP-RU) in conjunction with anti-CD3 antibody. As illustrated by the representative dot plots acquired via flow cytometry, MAIT cells were identified as CD3^+^ MR1-5-OP-RU tetramer^+^ lymphocytes. The frequency of MAIT cells was detected by flow cytometry. (We would like to thank Drs. James McCluskey, Jamie Rossjohn, and David Fairlie for their joint development of the MR1 tetramer technology.) **Figure 1B** presents a representative flow cytometry gating strategy for evaluating MAIT cell frequency within peripheral blood lymphocytes (PBL), utilizing MR1-6-FP tetramers as a negative control. **Figure 1A** illustrates a T-SNE dimensionality reduction plot that delineates the distribution of T-cell subsets in peripheral blood from patients with SRLI. MAIT cell analysis was performed across four groups: HC, NSLI, NLIS, and the SRLI group. Comparative analysis of MAIT cell frequencies in peripheral blood across these groups demonstrated a significant reduction in the proportion of MAIT cells in the SRLI group relative to healthy controls (p<0.0001). However, no significant difference was observed between the SRLI group and either the NSLI group or the NLIS group (**Figure 1C**).

As a population of unconventional T cells, MAIT cells in humans predominantly consist of the CD8^+^ subset, while the CD4^-^CD8^-^ (double-negative, DN) subset constitutes approximately 10%, and the CD4^+^ subset represents only a minor fraction. Different subsets may fulfill distinct functional roles. Therefore, this study analyzed the proportional distribution of MAIT cell subsets in peripheral blood across four groups: HC (n=40), NSLI (n=21), NLIS (n=13), and SRLI (n=33). **Figure 1D** depicts the identification of the subsets based on CD4 and CD8 staining following gating on MAIT cells. The results indicated that, across all groups, CD8^+^ MAIT cells were the predominant subset, followed by DN MAIT cells, with CD4^+^ MAIT cells being the least frequent. Compared with healthy controls, patients with SRLI exhibited a reduced proportion of CD8^+^ MAIT cells (p<0.01) and an increased proportion of CD4^+^ MAIT cells (p<0.05). In contrast, the proportion of DN MAIT cells did not show significant variation among the groups (**Figure 1D**). These findings suggest that the composition of peripheral MAIT cell subsets is altered in patients with SRLI.

In this study, correlation analyses were conducted between MAIT cell frequency and both SOFA and APACHE II scores in patients with SRLI. The results revealed a significant negative correlation between MAIT cell frequency and SOFA score (r=–0.5184, p=0.0047) as well as APACHE II score (r=–0.4645, p=0.0128) (**Figure 1E**), indicating that MAIT cell frequency declines with increasing disease severity.

### 3.2 Alterations in phenotype and function of peripheral blood MAIT cells in patients with SRLI

**Figure 2:**
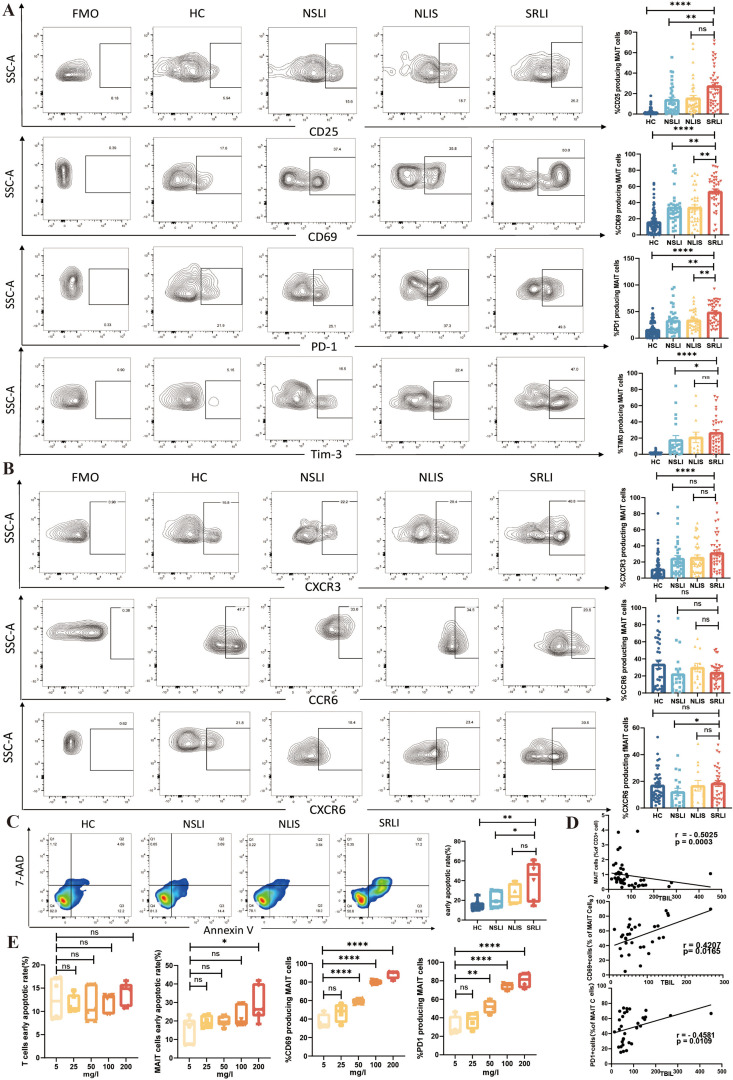
Phenotypic and functional changes of peripheral blood MAIT cells in patients with sepsis−induced liver injury. **(A)** Representative flow cytometry plots and statistical analysis of the expression of CD25, CD69, PD−1, and Tim−3 on peripheral blood MAIT cells across groups: HC (n=40−85), NSLI (n=18−34), NLIS (n=13−37), SRLI (n=31−40). **(B)** Representative flow cytometry plots and statistical analysis of the expression of chemokine receptors CXCR3, CCR6, and CXCR6 on peripheral blood MAIT cells across groups: HC (n=33−91), NSLI (n=18−34), NLIS (n=13−37), SRLI (n=31−39). **(C)** Representative flow cytometry plots and statistical analysis of apoptosis in peripheral blood MAIT cells across groups: HC (n=8), NSLI (n=5), NLIS (n=5), SRLI (n=6). **(D)** Spearman correlation analysis between MAIT cell frequency, expression levels of CD69 and PD−1 on MAIT cells, and serum total bilirubin levels in patients with sepsis−induced liver injury. **(E)** Apoptosis levels in T cells and MAIT cells from healthy donor peripheral blood PBMCs (n=6) after stimulation with different concentrations of bilirubin, as well as expression of CD69 and PD−1 on MAIT cells following stimulation with different concentrations of total bilirubin. Ns, not statistically significant; *p<0.05, **p<0.01, ***p<0.001, and ****p<0.0001, indicate statistical significance. HC, Health control; NSLI, Non-septic acute liver injury; NLIS, Non-associated liver injury in sepsis; SRLI, Sepsis-related liver injury.

MAIT cells are significant immunomodulatory cells that undergo a series of alterations upon activation, leading to diverse biological effects. Prior research has demonstrated that MAIT cells become activated in disease states, as indicated by increased surface expression of activation markers CD69 and CD25 (25). Concurrently, research has also demonstrated MAIT cell functional exhaustion, indicated by increased expression of immune checkpoint markers PD-1 and Tim-3 (26). To further investigate phenotypic changes in peripheral blood MAIT cells across four groups—HC (n=40-85), NSLI (n=18-34), NLIS (n=13-37), and SRLI (n=31-40)—this study employed flow cytometry. Following the gating of MAIT cells, fluorescence-minus-one (FMO) controls were employed to delineate positive populations for various activation and exhaustion markers, and the proportion of MAIT cells expressing each marker was subsequently analyzed. **Figure 2A** illustrates the gating strategy: upon identifying MAIT cells, FMO controls were utilized to define populations expressing CD25^+^, CD69^+^, PD-1^+^, and Tim-3^+^, followed by an analysis of their proportions within the MAIT cell population. The results indicated that in patients with SRLI, CD69 expression on peripheral blood MAIT cells was significantly upregulated compared to all other groups. CD25 expression was elevated relative to the HC and NSLI groups, although no statistically significant difference was observed when compared to the NLIS group. PD-1 expression was found to be upregulated in comparison to all other groups. Similarly, Tim-3 expression was higher relative to the HC and NSLI groups, but no significant difference was noted when compared to the NLIS group. These findings suggest that peripheral blood MAIT cells in patients with SRLI exhibit a considerable state of activation and exhaustion.

MAIT cells express several chemokine receptors that are vital for their migration to inflammatory sites. CXCR3 is a chemokine receptor that is highly expressed on activated T cells, whereas CCR6 and CXCR6 are closely associated with hepatic homing. These receptors play critical roles in T cell distribution, migration, and function (27). Therefore, this study aimed to investigate the migratory potential of MAIT cells in patients with SRLI toward inflammatory sites. **Figure 2B** illustrates the gating strategy employed for the identification of CXCR3^+^, CCR6^+^, and CXCR6^+^ populations within the MAIT cell subset, utilizing fluorescence-minus-one (FMO) controls, along with the statistical analysis of their proportions across different groups. Analysis of chemokine receptor expression on peripheral blood MAIT cells in the four groups—HC (n=33−91), NSLI (n=18−34), NLIS (n=13−37), and SRLI (n=31−39)—revealed that in the SRLI group, CXCR3 expression was significantly upregulated compared to the HC group (p<0.0001). However, no statistically significant differences were observed in the other receptors. Consequently, our data do not robustly support the hypothesis of enhanced hepatic migration of peripheral blood MAIT cells in this context.

Given the significant reduction in frequency and the hyperactivated/exhausted state of peripheral blood MAIT cells in patients with SRLI, and considering apoptosis as a predominant mechanism of cell death, we further investigated the apoptosis of MAIT cells in patients from the HC (n=8), NSLI (n=5), NLIS (n=5), and SRLI (n=6) groups.

PBMCs were initially gated for the identification of MAIT cells. Subsequently, these cells were subjected to staining with 7-AAD and Annexin V, utilizing an apoptosis detection kit to determine the percentage of 7-AAD^-^ Annexin V^+^ MAIT cells. **Figure 2C** illustrates a representative flow cytometry plot, while **Figure 2D** presents the corresponding statistical results. The results indicated that the proportion of apoptotic MAIT cells was significantly greater in patients with SRLI compared to HC and NSLI groups (p<0.05 and p<0.01, respectively). Conversely, no statistically significant difference was found between SRLI patients and those with NLIS.

### 3.3 High concentrations of total bilirubin (TBIL) can induce apoptosis in MAIT cells and promote their TCR-dependent activation

To investigate the causes of apoptosis and the activation of MAIT cells in the peripheral blood of patients with SRLI, this study examined both hyperbilirubinemia and inflammatory environments. Initially, the decreased frequency of MAIT cells in the peripheral blood of SRLI patients suggested a potential association between MAIT cells and liver injury. Therefore, we analyzed the correlation between the frequency of MAIT cells and clinical indicators of liver function. The results indicated that in these patients, the total bilirubin level was significantly negatively correlated with the frequency of peripheral blood MAIT cells, while the frequencies of CD69^+^ and PD-1^+^ MAIT cells exhibited a significant positive correlation with the total bilirubin level (**Figure 2D**).

Utilizing PBMCs from healthy donors (n=6), we assessed the impact of various concentrations of total bilirubin on MAIT cell apoptosis under *in vitro* stimulation. Significant apoptosis of MAIT cells was observed only after 24 h of exposure to 200mg/L (equivalent to 342 μmol/L) total bilirubin (p<0.05), whereas conventional CD3^+^ T cells demonstrated minimal changes in apoptosis (**Figure 2E**).

Subsequently, following a 24-hours co-culture of healthy donor PBMCs with total bilirubin, 5-OP-RU/MR1-loaded artificial antigen-presenting cells (aAPCs) were introduced to the culture system for an additional 24 h to stimulate the cells. The activation marker CD69 and the exhaustion marker PD-1 on MAIT cells were subsequently assessed. The results revealed a significant increase in the proportions of CD69^+^ and PD-1^+^ MAIT cells following TCR-dependent stimulation (**Figure 2E**).

### 3.4 Abnormal activation of the RORγt/IL-17 pathway in peripheral blood MAIT cells drives IL-17A-mediated organ dysfunction in SRLI

To examine alterations in transcription factors within peripheral blood MAIT cells derived from patients with SRLI, PBMCs were isolated from four distinct cohorts: HC (n=23), NSLI (n=11), NLIS (n=14), and SRLI (n=12). Initially, PBMCs were stained for surface markers, followed by fixation and permeabilization utilizing a nuclear fixation/permeabilization kit. Subsequently, cells were stained with fluorochrome-conjugated antibodies targeting specific transcription factors to evaluate alterations in nuclear transcription factor expression within MAIT cells.

**Figure 3:**
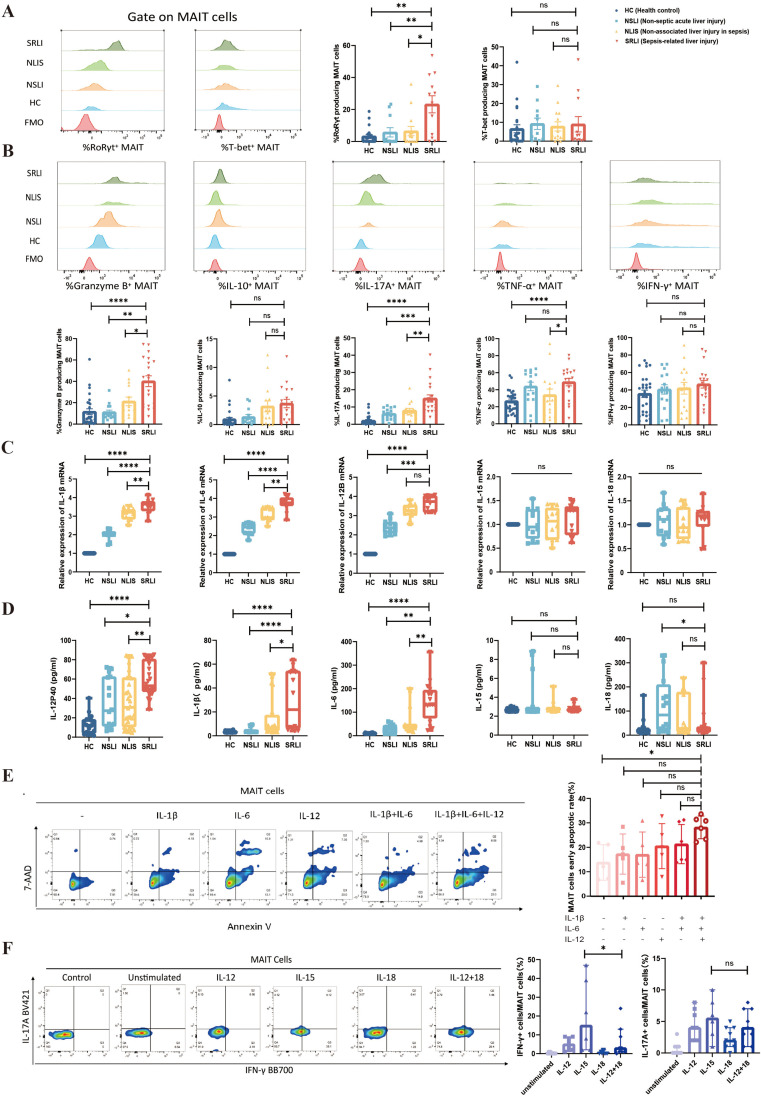
Impact of SRLI-related cytokines on peripheral blood MAIT cells. **(A)** Representative flow cytometry plots and statistical analysis of nuclear transcription factor T-bet and RORγt expression levels in peripheral blood MAIT cells across groups: HC (n=23), NSLI (n=11), NLIS (n=14), SRLI (n=12). **(B)** Intracellular cytokine secretion (Granzyme B, IL-10, IL-17A, TNF-α, IFN-γ) in peripheral blood MAIT cells across groups: HC (n=28), NSLI (n=13–15), NLIS (n=15), SRLI (n=18–20). **(C)** mRNA expression levels of IL-1β, IL-6, and IL-12p40 in peripheral blood PBMCs (n=20 per group) measured by qPCR. **(D)** Serum cytokine levels (IL-1β, IL-6, IL-12p40, IL-15, IL-18) measured by ELISA in each group (n=20 per group). **(E)** Apoptosis levels of MAIT cells in healthy donor peripheral blood PBMCs after 24-hour *in vitro* stimulation with IL-1β+IL-6+IL-12. **(F)** Intracellular cytokine secretion (IFN-γ and IL-17A) by MAIT cells in healthy donor peripheral blood PBMCs after 24-hour *in vitro* stimulation with IL-15. Ns, not statistically significant; *p<0.05, **p<0.01, ***p<0.001, and ****p<0.0001, indicate statistical significance. HC, Health control; NSLI, Non-septic acute liver injury; NLIS, Non-associated liver injury in sepsis; SRLI, Sepsis-related liver injury.

Flow cytometric analysis indicated that, in comparison to HC, patients with NSLI, and NLIS, the proportion of MAIT cells expressing retinoic acid receptor–related orphan receptor γt (RORγt) was significantly elevated in individuals with SRLI (p<0.05, p<0.001, p<0.001, respectively). Conversely, the proportion of MAIT cells expressing T-bet did not demonstrate significant differences among the groups (**Figure 3A**). Given that RORγt serves as the primary transcription factor for the Th17 cell lineage, these findings imply that peripheral blood MAIT cells in SRLI patients may exhibit a preferential skewing towards the Th17 (IL-17) pathway.

To further investigate the cytokine secretion profiles of MAIT cells, PBMCs from HC (n=28), NSLI (n=13-15), NLIS (n=15), and SRLI (n=18-20) groups were stimulated with PMA/ionomycin (PMA/ION), followed by intracellular cytokine staining. The results revealed that MAIT cells from sepsis patients secreted significantly higher levels of granzyme B, TNF-α, and IL-17A compared to healthy controls (p<0.0001 for all). Furthermore, in comparison to non-infectious acute liver injury patients, MAIT cells from SRLI patients exhibited markedly enhanced secretion of granzyme B and IL-17A (p<0.01 and p<0.001, respectively). Additionally, when compared to patients with NLIS, MAIT cells from SRLI patients also demonstrated significantly increased production of granzyme B, TNF-α, and IL-17A (p<0.0001 for all). There were no significant differences among groups in the secretion of IFN-γ or IL-10 by MAIT cells (**Figure 3B**).

Collectively, these data indicate that peripheral blood MAIT cells in SRLI patients likely secrete pro-inflammatory cytokines such as IL-17A through activation of the RORγt/IL-17 pathway.

### 3.5 Impact of cytokines in SRLI on MAIT cell function

To explore the cytokine profiles in SRLI, we quantified plasma levels of IL-1β, IL-6, IL-12p40, IL-15, and IL-18 through ELISA in patients with HC (n=20), NSLI (n=20), NLIS (n=20), and SRLI (n=20) groups. Additionally, the relative mRNA expression levels of these cytokines in PBMCs were evaluated using RT-qPCR. The findings indicated that plasma concentrations of IL-1β, IL-6, and IL-12p40 were significantly elevated in the group exhibiting SRLI compared to the other groups (**Figure 3C**). At the mRNA level, the expression of IL-1β and IL-6 was also significantly higher in this group (**Figure 3D**).

To further investigate the effects of the highly expressed inflammatory cytokines on peripheral blood MAIT cells in the context of SRLI, a standard concentration of 50 ng/mL was utilized. PBMCs isolated from healthy donors were cultured with IL-1β, IL-6, and/or IL-12 for a duration of 24 hours. MAIT cells were subsequently identified through flow cytometry, and apoptosis was detected using 7-AAD and Annexin V via an apoptosis assay kit. As shown in **Figure 3E**, significant apoptosis of MAIT cells was induced solely by the combination of IL-1β+IL-6+IL-12. Given that MAIT cells express abundant interleukin receptors such as IL-12R, IL-15R, and IL-18R, we also evaluated the impact of exogenous IL-12, IL-15, and IL-18 on cytokine secretion by MAIT cells.

The results demonstrated that IL-15 significantly enhanced the capability of MAIT cells to secrete IFN-γ, showing a statistically significant difference when compared to stimulation with the combination of IL-12 and IL-18 (p<0.05), whereas it did not significantly affect IL-17A secretion (**Figure 3F**). These findings suggest that IL-15 can directly stimulate MAIT cells to produce IFN-γ via a TCR-independent pathway.

### 3.6 Protective role of MAIT cells against LPS-induced SRLI in mice

**Figure 4:**
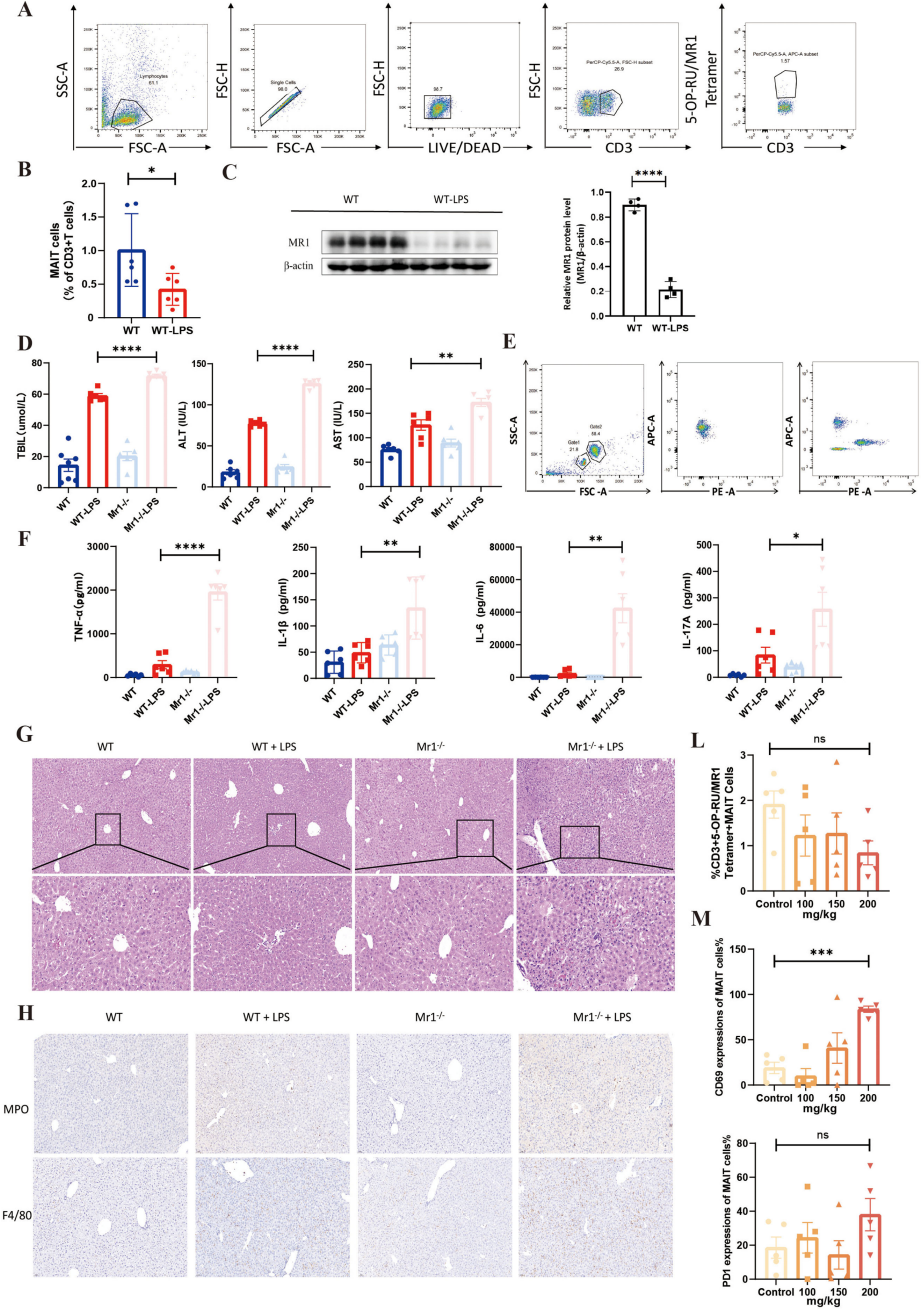
Protective role of MAIT cells in LPS−induced sepsis−associated liver injury in mice. **(A)** Gating strategy for identifying MAIT cells in single−cell suspensions from mouse liver tissue. **(B)** Frequency of MAIT cells in liver tissue from WT mice versus WT−LPS mice (6 vs 6). **(C)** MR1 expression levels in liver tissue of WT mice and WT−LPS mice detected by Western blot. **(D)** Serum levels of ALT, AST, and TBIL measured 24 h after induction of septic acute liver injury using LPS (or PBS as control) in WT and Mr1^−/−^mice. **(E)** Flow cytometry gating strategy for multiplex cytokine analysis in serum samples from each mouse group. **(F)** Serum levels of inflammatory cytokines (TNF−α, IL−1β, IL−6, and IL−17A) in WT, WT−LPS, Mr1^−/−^, and Mr1^−/−^−LPS mice 24 h after modeling. **(G)** H&E−stained liver sections from WT, WT−LPS, Mr1^−/−^, and Mr1^−/−^−LPS mice showing pathological changes. **(H)** Immunohistochemical detection of MPO and F4/80 expression in liver tissues from WT, WT−LPS, Mr1^−/−^, and Mr1^−/−^−LPS mice. **(L)** Frequency of MAIT cells in liver single−cell suspensions measured by flow cytometry 24 h after induction of hyperbilirubinemia in control, 100 mg/kg, 150 mg/kg, and 200 mg/kg total bilirubin−treated mouse groups. **(M)** Expression levels of CD69 and PD−1 on MAIT cells from mice treated with different doses of total bilirubin.Ns, not statistically significant; *p<0.05, **p<0.01, ***p<0.001, and ****p<0.0001, indicate statistical significance.

To analyze MAIT cells within liver tissue, fresh liver samples were obtained from both wild-type (WT) mice and WT mice subjected to LPS-induced SRLI (WT-LPS group). MAIT cells, defined in this study as CD3^+^ 5-OP-RU/MR1 tetramer^+^ lymphocytes, were identified through flow cytometry (the gating strategy is illustrated in **Figure 4A**). The results indicated that the proportion of MAIT cells in the livers of WT-LPS mice was significantly lower than that observed in untreated WT mice (p<0.05, **Figure 4B**). Furthermore, the expression level of MR1 in liver tissue from LPS-induced SRLI mice was markedly elevated compared to WT mice (p<0.0001, **Figure 4C**).

Subsequently, we investigated the influence of blocking MAIT cells on serum liver function parameters and inflammatory cytokine levels in mice with LPS-induced SRLI. Initially, WT-LPS and Mr1^-^/^--^LPS mice were intraperitoneally injected with LPS (10 mg/kg), while WT and Mr1^-^/^-^ (which are deficient in MAIT cells (28, 29)) control mice received an equivalent volume of PBS. Serum samples were collected from all experimental groups 24 h post-injection. Assessment of liver function indicators revealed that serum levels of ALT, AST, and TBIL in Mr1^-^/^--^LPS mice were significantly higher than those in WT-LPS mice (p<0.0001, p<0.01, p<0.0001, respectively; **Figure 4D**). Furthermore, serum concentrations of TNF-α, IL-1β, IL-6, and IL-17A were quantified using the LEGENDplex multiplex bead-based flow cytometry assay. **Figure 4E** presents the gating strategy for cytokine analysis, where each band within Gate1 and Gate2 corresponds to a specific cytokine. Following detection on a FACS Verse flow cytometer, standard curves were generated, and cytokine concentrations were analyzed using dedicated online analysis software. As depicted in **Figure 4F**, serum levels of TNF-α, IL-1β, IL-6, and IL-17A in Mr1^-^/^--^LPS mice were significantly elevated compared to the WT-LPS group (p<0.0001, p<0.01, p<0.01, p<0.05, respectively).

Histopathological analysis of liver tissue was performed 24 hours after the intraperitoneal injection of LPS (10 mg/kg). Liver tissues were harvested from each group, and pathological alterations were examined under a microscope following H&E staining. Representative H&E-stained liver sections from each group are illustrated in **Figure 4G**. In the WT and Mr1^-/-^ control groups, hepatocytes were organized in cords with distinct structure and normal morphology. Conversely, in the WT-LPS group, hepatic architecture was disorganized, exhibiting notable hepatocyte swelling and inflammatory cell infiltration. In the Mr1^-^/^--^LPS group, pronounced swelling and vacuolar changes were observed, accompanied by significant hepatocyte disintegration and death, as well as extensive inflammatory cell infiltration. Furthermore, immunohistochemical staining was performed on liver tissues to evaluate the distribution of MPO and F4/80. Representative images of MPO and F4/80 immunostaining in liver sections from each group are displayed in **Figure 4H**. The expression of both MPO and F4/80 was significantly elevated in the Mr1^-^/^--^LPS group compared to all other groups, indicating substantial activation of neutrophils and macrophages within the liver. These findings suggest that Mr1^-/-^ mice (lacking MAIT cells) experience more severe hepatic inflammatory cell infiltration in response to LPS-induced sepsis, implying that MAIT cells may serve a protective role in modulating excessive inflammatory responses in the liver during the early stages of sepsis.

Considering our previous findings indicated that high concentrations of total bilirubin can activate MAIT cells in a TCR-dependent manner *in vitro*, we further explored the effect of total bilirubin on hepatic MAIT cells *in vivo*. WT mice were divided into a control group and three treatment groups receiving 100 mg/kg, 150 mg/kg, or 200 mg/kg of total bilirubin to induce hyperbilirubinemia. Twenty-four hours post-treatment, fresh liver tissues were collected. A single-cell suspension of liver lymphocytes was generated using Percoll density gradient centrifugation. Hepatic MAIT cells were identified via flow cytometry, and subsequently, CD69^+^ MAIT cells and PD-1^+^ MAIT cells were gated within this population.

As illustrated in **Figure 4L**, the frequency of hepatic MAIT cells did not exhibit significant differences between the total bilirubin-treated groups and the control group. However, further analysis revealed that the proportion of CD69^+^ MAIT cells was significantly increased in the 200 mg/kg total bilirubin group compared to the control, while no differences were observed in the other treatment groups. The expression level of the exhaustion marker PD-1 did not demonstrate statistically significant differences among the groups (**Figure 4M**). This animal experiment corroborates that a high-concentration total bilirubin environment *in vivo* can significantly enhance the expression of the activation marker CD69 on hepatic MAIT cells.

## 4 Discussion

Sepsis is a life-threatening syndrome characterized by early hyperinflammation, prolonged immunosuppression, and sequential organ dysfunction. It is a significant cause of mortality among critically ill patients worldwide, and it also negatively impacts the quality of life for survivors (30) The pathogenesis of sepsis encompasses multiple stages, including pathogen recognition, inflammatory response, and immune reactions. Notably, the dysregulation of the immune response during sepsis is a significant contributor to the associated mortality (31). In individuals and conditions characterized as “sepsis-susceptible”, infection initiates a highly intricate inflammatory response that exhibits variability in its pro-inflammatory and anti-inflammatory components, contingent upon factors such as pathogen load and virulence, genetic factors, and other host determinants (32). The pro-inflammatory response observed in septic patients contributes to microbial eradication; however, it may subsequently result in organ damage. Conversely, anti-inflammatory and immunosuppressive mechanisms promote tissue repair but also increase the susceptibility of patients to secondary infections and opportunistic pathogens. Given the liver’s central role in immunity and its vulnerability to dysregulated inflammation during sepsis (33, 34), maintaining immune homeostasis is critical in SRLI. This study reveals that both the quantity and function of MAIT cells are significantly altered in patients with SRLI. The study aims to investigate the complex regulatory role of MAIT cells in disrupting local hepatic immune homeostasis during the development and progression of SRLI. By doing so, it seeks to identify potential therapeutic targets for this condition.

MAIT cells are a subset of T cells with immunomodulatory functions. They initiate innate immune responses upon recognizing MHC class I-related protein 1 (MR1) that presents small vitamin B metabolites (35). Recent studies have highlighted the significant role of MAIT cells in the onset and progression of sepsis. These cells can recognize antigens from pathogens and exert immunomodulatory effects, primarily through cytokine production and other mechanisms (35). In septic patients, the frequency of peripheral blood MAIT cells is significantly reduced, and the degree of this reduction is correlated with an elevated risk of acquiring infections in the intensive care unit (36). Reduced peripheral blood MAIT cell frequencies have also been reported in patients with inflammatory, autoimmune diseases, as well as in those with cancer (37–39). Similarly, in alcohol-related liver disease, such alterations correlate with liver injury severity (40). In the present study, we observed a significant decrease in the frequency of peripheral blood MAIT cells in patients with SRLI. The APACHE II score encompasses acute physiology, age, and chronic health subscores, with higher values indicating more severe disease, poorer prognosis, and increased mortality risk. The Sequential Organ Failure Assessment (SOFA) score, introduced in 1994 by the European Society of Intensive Care Medicine (ESICM), possesses inherent advantages for prognostic evaluation in sepsis as defined by Sepsis-3.0. Correlation analysis with clinical severity scores revealed a negative association between MAIT cell frequency and both SOFA and APACHE II scores. This finding suggests that MAIT cells may serve as potential indicators for assessing disease severity in SRLI; however, larger sample sizes are necessary to validate their utility as clinical biomarkers.

Based on the expression of CD4 and CD8 co-receptors, human circulating MAIT cells are primarily categorized into CD8^+^ and CD4^-^CD8^-^ (double-negative, DN) subsets, with CD4^+^ MAIT cells being less frequent. In our study, the proportion of CD4^-^CD8^+^ MAIT cells was significantly lower in patients with SRLI compared to healthy controls, whereas the proportion of CD4^+^CD8^-^ MAIT cells was notably increased. We hypothesize that the expanded CD4^+^ MAIT subset exerts immunomodulatory functions distinct from those of CD8^+^ MAIT cells, potentially reflecting the specific immune status of SRLI patients. Nevertheless, confirming this hypothesis requires additional data and theoretical support. Given their typically low frequency and the general neglect of this population in previous research, the CD4^+^ MAIT cell subset merits further investigation. Future studies will aim to elucidate the role of CD4^+^ MAIT cells in patients with SRLI.

MAIT cells can be activated by either microbial-derived metabolites (a TCR-dependent mode) or cytokines (a TCR-independent mode) (37). Upon activation, MAIT cells undergo a series of functional alterations, with a substantial body of research corroborating the upregulation of surface activation markers, including CD69 and CD25, across various pathological conditions. During early sepsis, MAIT cells upregulate activation (CD25, CD69, CD38, CD137, HLA-DR) and exhaustion markers (Tim-3, LAG-3) (1, 36, 41–43). In critically ill COVID-19 patients, increased CD69 expression on MAIT cells has been closely associated with worse clinical outcomes (42). Compared to non-MAIT CD3^+^ T cells, MAIT cells exhibit earlier and more pronounced upregulation of CD25 and CD69 (43). Once upregulated, CD69 expression can enhance cellular proliferation, cytokine production, and cytotoxic activity. Immune checkpoint molecules such as PD-1 and Tim-3, when upregulated, impair T cell activation and proliferation, leading to functional exhaustion and apoptosis (44, 45). A study identified that MAIT cells obtained from septic patients displayed a unique inhibitory receptor profile characterized by low levels of PD-1 and elevated expression of TIM-3, both of which are associated with T cell exhaustion (46). Our findings indicate that peripheral blood MAIT cells in patients with SRLI demonstrate upregulated expression of both activation and exhaustion markers, suggesting an altered functional state consistent with post-activation exhaustion. Furthermore, our results reveal increased apoptosis in MAIT cells from these patients, correlating with the observed elevations in PD-1 and TIM-3 expression. Therefore, we propose that the heightened levels of activation and exhaustion markers on peripheral blood MAIT cells in patients with SRLI reflect activation-induced cell death, a mechanism that may serve to regulate uncontrolled inflammation and mitigate further damage to healthy tissues.

Additionally, MAIT cells express chemokine receptors such as CCR6 and CXCR3, which facilitate their tissue homing and may elucidate their redistribution in inflammatory diseases (47, 48). Several studies have demonstrated that MAIT cells can migrate to infected tissues, thereby decreasing circulating frequency that recovers after treatment (49). MAIT cells express CCR6 (binding CCL20 in liver/mucosa) and CXCR6 (binding CXCL16 on liver sinusoidal endothelial cells) (48, 50, 51), which facilitate hepatic homing and may explain their enrichment in the human liver. Hepatic MAIT cells resemble their circulating counterparts but display local activation features such as high CD69 expression, promoting tissue retention (52). In the present study, we observed a significantly elevated expression of CXCR3 on peripheral blood MAIT cells from patients with SRLI in comparison to other groups. However, the levels of the chemokine receptors CCR6 and CXCR6 on MAIT cells were not markedly upregulated. These findings do not provide robust evidence to support the migration of MAIT cells to the liver in this context. Nevertheless, due to the absence of patient liver samples, the specific genetic expressions, signaling pathways, and additional factors influencing MAIT cell migration to the liver necessitate further in-depth investigation to elucidate the underlying mechanisms more comprehensively.

Murine MAIT cells are categorized into developmentally, transcriptionally, and functionally distinct subsets: MAIT1, MAIT17, and the proposed developmental intermediate MAIT2 (53). In contrast, human MAIT cells do not exhibit distinct MAIT1 and MAIT17 subset characteristics. Instead, their functional profiles align more closely with those of Th1 and Th17 cells leading to their classification as Th1-like and Th17-like MAIT cells (54), which exert protective roles in various infectious diseases through the secretion of distinct cytokines. Th1-like MAIT cells primarily mediate cellular immune responses through the secretion of cytokines such as IFN-γ and TNF-α, which serve to activate macrophages and enhance their pathogen-killing and phagocytic capabilities. Conversely, Th17-like MAIT cells secrete inflammatory cytokines like IL-17 and IL-22, thereby contributing to the defense against autoimmune diseases, host defense responses, and inflammatory processes. Notably, MAIT cells execute their effector functions under the regulation of distinct transcription factor patterns, which include the type 1 transcription factor T-bet, the type 2 factor promyelocytic leukemia zinc finger (PLZF), and the type 17 factor RORγt (55). MAIT1 (T-bet^+^) and MAIT17 (RORγt^+^) subsets bias cytokine production toward IFN-γ or IL-17A, respectively (56). Our study demonstrates that MAIT cells derived from patients with SRLI secrete significantly elevated levels of IL-17A. However, the precise role of IL-17A in this context remains inconclusive. IL-17A may facilitate hepatic inflammatory responses and stimulate the production of additional cytokines, thereby contributing to liver dysfunction. Additionally, IL-17A has the potential to induce neutrophil infiltration into the liver, which may further exacerbate inflammation and liver injury. Multiple studies have reported an increased frequency of IL-17A^+^ MAIT cells in inflammatory conditions (57), which is consistent with our findings of significantly elevated proportions of both IL-17A^+^ MAIT cells and RORγt^+^ MAIT cells in the peripheral blood of sepsis−induced liver injury patients. Although MAIT17 cells differ from Th17 cells regarding tissue retention regulation and innate/cytotoxic gene expression, the key transcriptional regulators governing IL-17A expression are conserved between MAIT17 and Th17 cells. Therefore, we hypothesize that peripheral blood MAIT cells in SRLI patients may manifest a Th17-skewed phenotype, suggesting that Th17-like MAIT cells could play a role in the pathogenesis of sepsis-associated liver injury. Then, an apparent paradox in our findings is that MAIT cells from patients with sepsis-associated liver injury exhibit a Th17-like phenotype (increased RORγt expression and IL-17A production), yet our *in vivo* data demonstrate that MAIT cells protect against LPS-induced liver injury by suppressing excessive inflammation. This apparent contradiction can be reconciled by considering the context-dependent functions of IL-17A-producing lymphocytes. Recent studies have established that IL-17A plays a dual role in sepsis and inflammatory conditions: while excessive, dysregulated IL-17A can contribute to tissue damage, controlled production by innate-like T cells such as γδ T17 cells and MAIT cells mediates protective antimicrobial defense and tissue repair (58–60). Notably, the controlled production of IL-17A by γδ T17 cells has been demonstrated to prevent SRLI (59). Furthermore, MAIT cells exhibit remarkable functional plasticity, adapting their effector profiles to microenvironmental cues. This plasticity ensures that MAIT17 cells play a predominant role in tissues, generating an antimicrobial MAIT1 response that contributes to the global MAIT1 pool during subsequent systemic infections (61). Thus, the Th17-like phenotype of MAIT cells in sepsis-associated liver injury should not be interpreted simply as a pro-inflammatory, pathogenic state. Critically, the absence of MAIT cells in our mouse model resulted in hyperinflammation (significantly elevated TNF-α, IL-1β, IL-6, IL-17A) and exacerbated liver injury, demonstrating that MAIT cells function to restrain excessive inflammatory responses. Moreover, in sepsis-associated liver injury, while the initial inflammatory response is essential for pathogen control, it is also crucial that it be tightly regulated to avoid collateral tissue damage. This bias toward a Th17-like phenotypic differentiation may thus represent a controlled local inflammatory program with multiple protective functions: enhancing antimicrobial defense at the site of infection, promoting tissue repair mechanisms, and preventing runaway systemic inflammation that leads to multiple organ dysfunction. Thus, we propose that the Th17-like MAIT cell response in sepsis-associated liver injury reflects a controlled effector program that balances antimicrobial defense with tissue protection, consistent with the established protective role of MAIT cells in SRLI. Importantly, we observed a notable upregulation of the cytotoxic marker granzyme B in peripheral blood MAIT cells from SRLI patients. Granzyme B is capable of targeting and eliminating pathogens *in vivo*, thereby exerting an anti-infective effect.

Compared to healthy individuals, septic patients exhibit elevated plasma levels of IL-1β, IL-6, IL-8, IL-10, IL-17, and TNF-α (62). Among these, plasma IL-1β levels are increased in non-survivors and show a progressive rise over time (63). Classical interleukins serve multifaceted roles in the inflammatory cascade, with sepsis-induced liver dysfunction recognized as a significant inflammatory response associated with disease severity. MAIT cells can be activated in a TCR-independent manner by a range of cytokines, including IL−12, IL−18, and IL−15 (39). Once activated, MAIT cells in turn produce a range of cytokines, such as IFN−γ, IL−17A, and granzyme B, to coordinate immune responses (64). Our results demonstrated that patients with SRLI exhibited significantly elevated concentrations of IL-1β, IL-6, and IL-12p40 compared to other groups. We further investigated the impact of IL-12, IL-15, and IL-18 on the cytokine-secreting capacity of MAIT cells. Our findings indicated that IL-15 significantly enhanced the ability of MAIT cells to secrete IFN-γ, while exerting no marked effect on IL-17A production, suggesting that IL-15 can directly stimulate MAIT cells to generate IFN-γ. Moreover, the highly expressed inflammatory cytokines IL-1β, IL-6, and IL-12 in the SRLI were shown to markedly induce MAIT cell apoptosis. This observation implies that MAIT cells may facilitate the elimination of hyper-reactive cells under high-inflammatory conditions, contributing to the maintenance of immune system homeostasis. This hypothesis is further corroborated by animal experiments, which revealed that MAIT-cell-deficient septic mice exhibited significantly elevated serum concentrations of TNF-α, IL-1β, IL-6, and IL-17A compared to WT septic mice. Additionally, we examined the effect of MR1-dependent MAIT cells on neutrophils and macrophages in the livers of septic mice. The results indicated that, in the murine sepsis model, MAIT-cell-deficient mice developed more severe liver injury and exhibited significantly greater numbers of neutrophils and macrophages in the liver compared to WT mice. Hence, we propose that MAIT cells may play a protective role in SRLI by modulating cytokine expression and maintaining the balance of the immune response, thereby mitigating tissue damage.

In critically ill septic patients, cholestasis is one of the primary causes of hyperbilirubinemia. Hyperbilirubinemia in sepsis can arise from extrahepatic biliary obstruction due to conditions such as choledocholithiasis or cholangitis, as well as from an increased bilirubin load secondary to trauma or hemolysis (15). Notably, clinical symptoms such as cholestasis and jaundice in septic patients can strongly elevate the risk of mortality, partly due to complications including intestinal bacterial translocation, gastrointestinal dysfunction, and multi-organ failure (65). In this study, we observed a significant correlation between the activation marker CD69 and the exhaustion marker PD-1 on peripheral blood MAIT cells, as well as serum total bilirubin levels in patients with SRLI. Consistent with this finding, exposure to 50mg/L total bilirubin significantly enhanced both the activation and exhaustion levels of MAIT cells following stimulation with artificial antigen-presenting cells (aAPCs). *In vivo* experiments in murine models further confirmed that elevated concentrations of total bilirubin promote the activation of hepatic MAIT cells. Our study is first investigation into the relationship between liver function and MAIT cells in SRLI, and reveals novel findings. Further mechanistic investigations are warranted to comprehensively examine the effects of various forms of bilirubin on MAIT cells, both *in vitro* and *in vivo*.

Key limitations of this study include the lack of paired human liver tissue samples, which prevented direct comparison of MAIT cell phenotypes between the patient’s circulation and the target organ. However, obtaining such paired samples presents substantial practical challenges, as liver biopsy is not a routine diagnostic or therapeutic procedure in ICU patients with sepsis−induced liver injury, informed consent from both patients and families is difficult to secure, the biopsy itself carries inherent risks, and critically ill patients often exhibit unstable and rapidly evolving clinical conditions. Second, in the critical care setting, although we assessed concomitant medication use, it remains challenging to fully dissociate the potential hepatotoxic effects of essential drugs (e.g., antibiotics, vasopressors) from sepsis-induced injury itself. This is an inherent limitation of observational clinical studies in this population. Third, only a MAIT cell-deficient model was employed. Although this study clearly demonstrates a protective role of MAIT cells in the pathogenesis of sepsis−associated liver injury *in vivo*, adoptive transfer studies will be required in the future to confirm sufficiency and evaluate direct therapeutic potential. Fourth, we acknowledge that the intraperitoneal LPS injection model used in this study represents an endotoxemia model rather than a true polymicrobial sepsis model. While it is well-established for investigating acute inflammatory liver injury, it does not capture the full complexity of clinical sepsis. Future studies using more translationally relevant models such as cecal ligation and puncture (CLP) are warranted to validate our findings. In addition, while the observed activated/exhausted phenotype is consistent with tissue migration or response to inflammatory signals, it has not been definitively confirmed by the available data. Finally, further mechanistic investigation has been limited by the technical difficulty of isolating and culturing primary MAIT cells. Thus, future studies should incorporate patient liver biopsies and related mechanistic approaches to fully address these gaps.

In summary, the discovery and investigation of MAIT cells have unveiled a significant component of the human innate-like immune system, which plays a crucial role in the rapid response and defense against pathogen invasion. MAIT cells are capable of swiftly recognizing and eliminating infected cells and pathogens, thereby curtailing the further dissemination of infection. In the context of sepsis, peripheral blood MAIT cells can modulate the inflammatory response through various mechanisms, such as functional activation and cytokine secretion, thereby ultimately mitigating additional hepatic damage. Although the role of MAIT cells in SRLI has been elucidated, future studies are needed to investigate the specific mechanisms underlying their actions in this condition. Additional investigation is also required to explore how modulation of MAIT cell function could potentially enhance therapeutic outcomes for sepsis-associated liver injury.

## Data Availability

The raw data supporting the conclusions of this article will be made available by the authors, without undue reservation.

## Supplementary material

Supplementary Table 1 Health controls baseline characteristics.

Supplementary Table 2 Microorganisms isolated from the blood of patients with septic liver injury.

Supplementary Table 3 Primers used in this study.

Supplementary Table 4 The clones, suppliers, and catalog numbers of all antibodies used in the study.
