# Supplementary material for: Protective role of mucosa-associated invariant T cells in sepsis-related liver injury
Source: Front Immunol. 2026 Apr 15;17:1779656. doi: 10.3389/fimmu.2026.1779656 (PMC13125037; doi:10.3389/fimmu.2026.1779656)
Supplement: Supplementary Table 1 — Health controls baseline characteristics. [file Table1.docx]

| **Supplemental Table 1. Health Controls Baseline Characteristics** | |
| --- | --- |
|  | **Health Control** |
| Age , y | 50.00 [37.00, 59.00] |
| Male sex , n (%) | 65 (56.52%) |
| Height , cm | 167.00 [162.00, 174.00] |
| Weight , kg | 63.00 [56.50, 73.00] |
| White blood cell (WBC) , 109/L | 5.89 [4.81, 6.94] |
| Neutrophils percentage (NEUT% ) , % | 55.26 [50.9, 59.2] |
| Lymphocyte (LYMPH) , 109/L | 2.04 [1.70, 2.52] |
| Hemoglobin (HGB) , g/L | 145.32 [135.0, 154.0] |
| Platelet (PLT) , 109/L | 229.00 [182.50, 272.00] |
| Albumin (ALB) , g/L | 45.40 [43.40, 46.95] |
| Total bilirubin (TBil) , μmol/L | 13.31 [10.35, 17.00] |
| Direct Bilirubin (DBil) , μmol/L | 4.30 [3.30, 5.65] |
| Indirect bilirubin (IBil) , μmol/L | 9.20 [7.40, 11.10] |
| Alanine aminotransferase‌ (ALT) , U/L | 21.00 [15.50, 29.50] |
| Aspartate transaminase (AST) , U/L | 20.00 [16.00, 25.50] |
