# Supplementary material for: Protective role of mucosa-associated invariant T cells in sepsis-related liver injury
Source: Front Immunol. 2026 Apr 15;17:1779656. doi: 10.3389/fimmu.2026.1779656 (PMC13125037; doi:10.3389/fimmu.2026.1779656)
Supplement: Supplementary Table 2 — Microorganisms isolated from the blood of patients with septic liver injury. [file Table2.docx]

| **Supplemental Table 2. Microorganisms isolated from the blood of patients with septic liver injury** | |
| --- | --- |
|  | **Blood culture results , n (%)** |
| Positive culture results | 43 (92.5) |
| Klebsiella pneumoniae | 8 (17.0) |
| Acinetobacter baumannii | 9 (19.1) |
| Pseudomonas aeruginosa | 5 (10.6) |
| E.coli | 3 (6.4） |
| Enterococcus faecalis | 1 (2.1) |
| Enterobacter cloacae | 1 (2.1) |
| Pseudomonas putida | 5 (1.1) |
| Stenotrophomonas maltophilia | 4 (8.5) |
| Staphylococcus aureus | 3 (6.4) |
| Burkholderia cepacia | 1 (2.1) |
| Pseudomonas tropicalis | 3 (6.4) |
| Others | 7 (14.9) |
| Negative culture results | 4 (7.5) |
