# Supplementary material for: Protective role of mucosa-associated invariant T cells in sepsis-related liver injury
Source: Front Immunol. 2026 Apr 15;17:1779656. doi: 10.3389/fimmu.2026.1779656 (PMC13125037; doi:10.3389/fimmu.2026.1779656)
Supplement: Supplementary Table 3 — Primers used in this study. [file Table3.docx]

| **Supplemental Table 3. Primers used in this study** | | |
| --- | --- | --- |
| **Gene** | **Forward primer (5’-3’)** | **Reverse primer (5’-3’)** |
| *IL-1β* | CCAAACCTCTTCGAGGCACA | GCTGCTTCAGACACTTGAGC |
| *IL-6* | CCGGGAACGAAAGAGAAGCTC | ACCGAAGGCGCTTGTGGAG |
| *IL-12B* | CCTGCCCAGAGCAAGATGTG | AGTTCCCATATGGCCACGAG |
| *IL-15* | TCCATCCAGTGCTACTTGTGT | CTGCACTGAAACAGCCCAAAA |
| *IL-18* | AGCTTCGGGAAGAGGAAAGG | AGCTTCGGGAAGAGGAAAGG |
| *GAPDH* | GGAGCGAGATCCCTCCAAAAT | GGCTGTTGTCATACTTCTCATGG |
