# Supplementary material for: Protective role of mucosa-associated invariant T cells in sepsis-related liver injury
Source: Front Immunol. 2026 Apr 15;17:1779656. doi: 10.3389/fimmu.2026.1779656 (PMC13125037; doi:10.3389/fimmu.2026.1779656)
Supplement: Supplementary Table 4 — The clones, suppliers, and catalog numbers of all antibodies used in the study. [file Table4.docx]

| **Supplemental Table 4. The clones, suppliers, and catalog numbers of all antibodies used in the study** | | | |
| --- | --- | --- | --- |
| **Anyibody** | **Vendors** | **Catalog Numbers** | **Clones** |
| APC-human-5-OP-RU | NIH TETRAMER CORE FACILITY | - | - |
| APC-human-6-FP | NIH TETRAMER CORE FACILITY | - | - |
| Anti-mouse-5-OP-RU/MRI-APC | NIH TETRAMER CORE FACILITY | - | - |
| Anti-human CD3-FITC | BD Pharmingen | 555332 | UCHT1 |
| Anti-human CD4-BV421 | BD Pharmingen | 562970 | SK3 |
| Anti-human CD8-PerCP-Cy5.5 | BD Pharmingen | 565310 | SK1 |
| Anti-human CD69-PerCP-Cy5.5 | BD Pharmingen | 560738 | FN50 |
| Anti-human PD1-PE | BD Pharmingen | 560795 | EH12.1 |
| Anti-human CD25-PE-CY7 | BD Pharmingen | 557741 | M-A251 |
| Anti-human Tim3-PE | BD Pharmingen | 563422 | 7D3 |
| Anti-human CXCR3-BV421 | BD Pharmingen | 562558 | 1C6/CXCR3 |
| Anti-human CCR6-PE-CY7 | BD Pharmingen | 560620 | 11A9 |
| Anti-human CXCR6-BV421 | BD Pharmingen | 566007 | 13B 1E5 |
| Anti-human IFN-γ-BB700 | BD Pharmingen | 566394 | B27 |
| Anti-human TNF-α-PE-CY7 | Biolegend | 502930 | MAb11 |
| Anti-human IL-10-PE | BD Pharmingen | 554706 | JES3-19F1 |
| Anti-human Granzyme B-BV510 | BD Pharmingen | 563388 | GB11 |
| Anti-human IL-17A-BV421 | BD Pharmingen | 562933 | N49-653 |
| Anti-human t-bet-BV421 | BD Pharmingen | 563318 | O4-46 |
| Anti-human Rorγt-PE | BD Pharmingen | 567532 | Q21-559 |
| Fixable Viability Stain 780 | BD Pharmingen | 565388 | - |
